# Efficient target control of complex networks based on preferential matching

**DOI:** 10.1371/journal.pone.0175375

**Published:** 2017-04-06

**Authors:** Xizhe Zhang, Huaizhen Wang, Tianyang Lv

**Affiliations:** 1 Key Laboratory of Medical Image Computing of Northeastern University, Ministry of education, Shenyang, Liaoning, China; 2 School of Computer Science and Engineering, Northeastern University, Shenyang, Liaoning, China; 3 College of Computer Science and Technology, Harbin Engineering University, Harbin, Heilongjiang, China; 4 IT Center, National Audit Office, Beijing, China; Nankai University, CHINA

## Abstract

Controlling a complex network towards a desired state is of great importance in many applications. Existing works present an approximate algorithm to find the input nodes used to control partial nodes of the network. However, the input nodes obtained by this algorithm depend on the node matching order and cannot achieve optimum results. Here we present a novel algorithm to find the input nodes for target control based on preferential matching. The algorithm elaborately arranges the matching order of the nodes to reduce the size of the input node set. The results on both synthetic and real networks indicate that the proposed algorithm outperforms the previous algorithm.

## Introduction

The control of complex networked systems plays an important role in many nature and technology applications. According to control theory [1–3], a system is controllable if the system can be driven from any initial state to any desired state in finite time. The external control signals can be inputted into the system through some suitable selected nodes. The nodes which received independent external signals are called input nodes [4], controls [5] or driver nodes[6]. An input node is the first node of a control path which transmits the control signals.

The input nodes, used to fully control the network, can be obtained by maximum matching of the network [7]. The unmatched nodes are the minimum set of input nodes (in short, *MIS*). Based on this framework, the researchers have analyzed the structural properties of *MIS* [8–10], roles of nodes in control [5], and robustness of controllability [11]. The size of *MIS* is found to be tied to the degree distribution [6], and mainly dominated by the number of the source and sink nodes [5]. Furthermore, the possible input nodes which participate in at least one *MIS* are connected by the input adjacency [4], and they exhibit a surprising bifurcation phenomenon of the dense networks [12], which is rooted by the emergence of giant control component [4].

In many real control scenarios, only a small fraction of nodes need to be controlled. This is called target control [13]. To control the target community of a network, Piao et.al [14] presented a method which used immune nodes to facilitate the control of target communities. To find the input nodes to control any specific target nodes, a recent work [13] presented an analysis framework to investigate the target control of complex networks. They proposed an approximate greedy algorithm (*GA*) based on multiple maximum matchings to obtain the input nodes used to control the target nodes.

**Fig 1:**
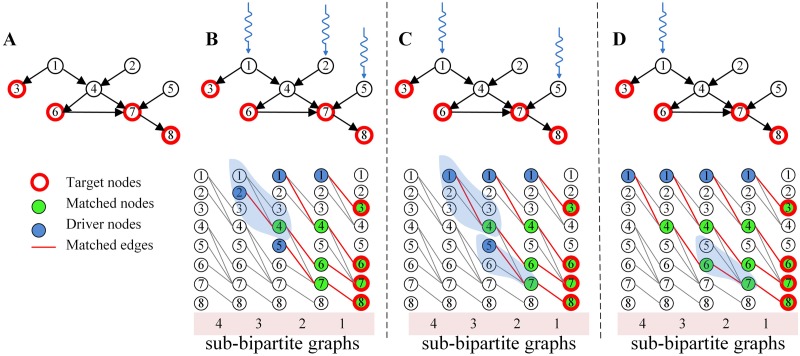
Illustration of random matching using *GA*. (A) A sample network *G* with target nodes {3, 6, 7, 8}; (B-D). Three *MIS*s obtained by *GA* and their matching process, in which *D*_1_ = {1, 2, 5}, *D*_2_ = {1, 5}, *D*_3_ = {1}. The input nodes are obtained by the following process: 1. Construct a bipartite graph *B* (sub-bipartite graph 1) in which the right side contains all target nodes and the left side contains the nodes pointing to the target nodes; 2. Find a maximum matching of *B* and denote the matched nodes by *M*; and 3. Let *M* be the set of new target nodes, and repeat steps 1 and 2 until no new matched nodes are found. The differences between the three matching processes are highlighted by the blue shadow.

However, the *GA* can only find the approximate minimum set of input nodes. If there exists more than one maximum matching in the network, the results of the *GA* strongly depend on which maximum matching is selected. For example, the number of input nodes may vary over a large range [13] (Fig 1). Therefore, finding the minimum number of input nodes for target control is still an unsolved problem.

Here, we present a novel algorithm for finding input nodes to control the target nodes of a network. In contrast to the previous approach, we elaborately arranged the matching order of the nodes and tried to reduce the total number of input nodes. The results on both synthetic and real networks showed that we obtained fewer input nodes than the previous approach.

## Method

Consider a linear time-invariant system, its states can be described by the following:

$$\{\begin{array}{c} \frac{\text{d}x}{\text{d}t}=Ax+Bu\\ y=Cx \end{array}$$ (1)

Where **x**(*t*) = (*x*_1_(*t*),…, *x*_*N*_(*t*))^T^ represents the system’s state; ***u***(*t*) = (*u*_1_(*t*),…,*u*_*M*_(*t*))^T^ represents the input vector and *y* represents the output vector; *A* is the transpose of the adjacency matrix, *B* is the input matrix and *C* is the output matrix which defines the target nodes we want to control. Let the network representation of above system be *G*(*V*, *E*), where *V* is the nodes set and *E* is the edges set. For a target node set *T*, we say the system is target controllable if the states of the target node set *T* can be driven from any initial state to a desired final state [13].

In previous work [13], a *k*-walk theory was proposed, and this theory proved that in a tree-like network, if a node has paths of different lengths to each target node, the node can control these target nodes. However, a single node cannot control all target nodes in many networks. Therefore, for more general networks with loops, previous work [13] proposed an approximate algorithm based on multiple maximum matchings to obtain the input nodes. The algorithm constructs a series of bipartite graphs *B* = {*B*_1_(*T*_1_,*F*_1_,*E*_1_),…,*B*_i_(*T*_*i*_,*F*_*i*_,*E*_*i*_)} by following procedures: 1. Let the set of target nodes be *T*_1_, find the set of in-neighbor nodes of *T*_1_ and denoted it as *F*_1_, construct bipartite graphs *B*_1_(*T*_1_,*F*_1_,*E*_1_), where *E*_1_ is the set of edges between nodes set *T*_1_ and *F*_1_;2. Let the *F*_1_ be the new target set *T*_2_ and repeat step 1 to get bipartite graph *B*_2_(*T*_2_,*F*_2_,*E*_2_); 3. Repeat above steps until the set of in-neighbor nodes of the current target set *T*_*i*_ is empty. After constructing the bipartite graphs, the algorithm finds the maximum matchings of each bipartite graph, and the union of the unmatched nodes of all bipartite graphs is the set of input nodes used to control the target nodes.

The key idea of this algorithm is to find the maximum matching for each sub-bipartite graph. However, in most networks, the maximum matchings are not unique. Therefore, even for a simple network, the algorithm produced different results with different maximum matchings. For example, for the network shown in Fig 1A, the algorithm obtained three different input node sets: *D*_1_ = {1, 2, 5}, *D*_2_ = {1, 5} and *D*_3_ = {1}. The reason for the multiple results is that the maximum matchings used in the algorithm are different. For example, if we matched edge *e*(1→4) rather than *e*(2→4) in sub-bipartite graph 3, node 2 would not act as an input node, resulting in the input node set *D*_2_ = {1, 5}. If we match edge *e*(6→7) rather than *e*(5→7) in sub-bipartite graph 2, we obtain only one input node *D*_1_ = {1} to control the entire target node set.

Therefore, to reduce the total number of input nodes, we need to select the appropriate maximum matching for each sub-bipartite graph. However, the number of unmatched nodes of each sub-bipartite graph is fixed because the maximum matchings of each bipartite graph have the same size. The only way to decrease the number of input nodes is to allow the input nodes of different sub-bipartite graphs to overlap with one another. For example, in Fig 1D, the unmatched node of all four sub-bipartite graphs is node 1, which decreases the total number of input nodes from three to one.

To obtain the expected input nodes of each bipartite graph, we use the preferential matching [15] to find maximum matching of each bipartite graph. The preferential matching method arranges the matching order of the nodes based on a predefined queue, and ensure that the nodes in the rear of the queue have a high probability of being input nodes. The preferential matching method first constructs a series of sub-graphs based on the node queue, and then finds the maximum matching of each sub-graph until the maximum matching of the whole network is obtained. This iterative matching process ensures that the nodes in the front of the queue have a high probability to be matched. Therefore, the resulted input nodes are most likely the nodes in the rear of the queue.

Therefore, the problem is selecting the appropriate input nodes of each sub-bipartite graph to reduce the total number of input nodes. Here we present the following strategies:

1. The input nodes of the current sub-bipartite graph should be overlapped with the input nodes of the previous sub-bipartite graph. This process will decrease the total number of input nodes.
2. The nodes that frequently appear in the matching graph (for all sub-bipartite graphs) should be input nodes with high priority, which will give the nodes in subsequent sub-bipartite graphs high probability to overlap with existing input nodes.

**Fig 2:**
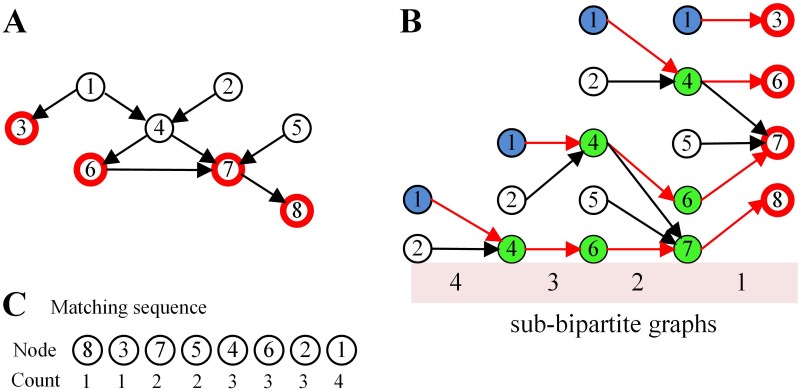
Illustration of preferential matching for target control. (A). A sample network *G* with target nodes {3, 6, 7, 8}. (B). Matching graph for target nodes {3, 6, 7, 8}. (C). Matching sequence of nodes based on their counts in the matching graph. The counts for node sequence {*n*_1_,*n*_2_,*n*_6_,*n*_4_,*n*_5_,*n*_7_,*n*_3_,*n*_8_} are {4,3,3,3,2,2,1,1}.

Fig 2 illustrates these strategies on an example network. For the network shown in Fig 2A, we construct a matching graph (*MG*) that starts from the target nodes and iteratively adds the parent nodes of current nodes to the graph, until no more nodes are added. We count the frequency with which each node appeared in the matching graph and arrange the nodes in ascending order of frequency. For example, Fig 2B shows the matching graph of Fig 2A, and the counts of nodes are *n*_1_ = 4, *n*_2_ = 3, *n*_3_ = 1, *n*_4_ = 3, *n*_5_ = 2, *n*_6_ = 3, *n*_7_ = 2 and *n*_8_ = 1, respectively. Therefore, the matching sequence of nodes should be {*n*_8_, *n*_3_, *n*_7_, *n*_5_, *n*_4_, *n*_6_, *n*_2_, *n*_1_} according their counts by ascending order. For each sub-bipartite graph of *MG*, we used this matching sequence to find input nodes.

Overall, for a network *G* and target node set *T*, the algorithm based on preferential matching (*PM*) for finding input nodes consists of the following steps:

1. For target node set *T*, construct bipartite graph B_1_(*F*, *T*), where *F* are the node sets pointing to target node set *T*.
2. Let *F* be the new target node set. Repeat step 1 to construct bipartite graph *B*_*i*_(*F*, *T*) until no more nodes are found. Define the matching graph *M*(*T*) = {*B*_1_, *B*_2_, …, *B*_*i*_}.
3. For each node in *M*, compute their counts *f*(*n*), arrange the nodes by ascending order of *f*(*n*) and let *Q* be the matching sequence.
4. For a sub-bipartite graph of *M*, find the maximum matching based on preferential matching using node sequence *Q*. Let *D* = {*d*_1_,*d*_2_,…,*d*_*i*_} be the set of input nodes. Rearrange the node sequence by putting the nodes of *D* in the rear of *Q*.
5. Repeat step 4. Find input nodes *D*_*i*_ of sub-bipartite graph *B*_*i*_. The final set of input nodes to control the target nodes is *D* = ∪ *D*_*i*_.

## Result

To quantify the efficiency of the algorithm, we evaluated the fraction of input nodes *n*_*D*_ = *N*_*D*_/*N* based on a *PM* algorithm and *GA* [13]. We used the following two different schemes for target node selection:

1. Random selection scheme: Select nodes from the network uniformly at random as targets, until reaching the expected target fraction *f*.
2. Local selection scheme: Randomly select a seed node, and expand the node based on a *breadth-first search* (BFS) tree, until reaching the expected target fraction *f*.

**Fig 3:**
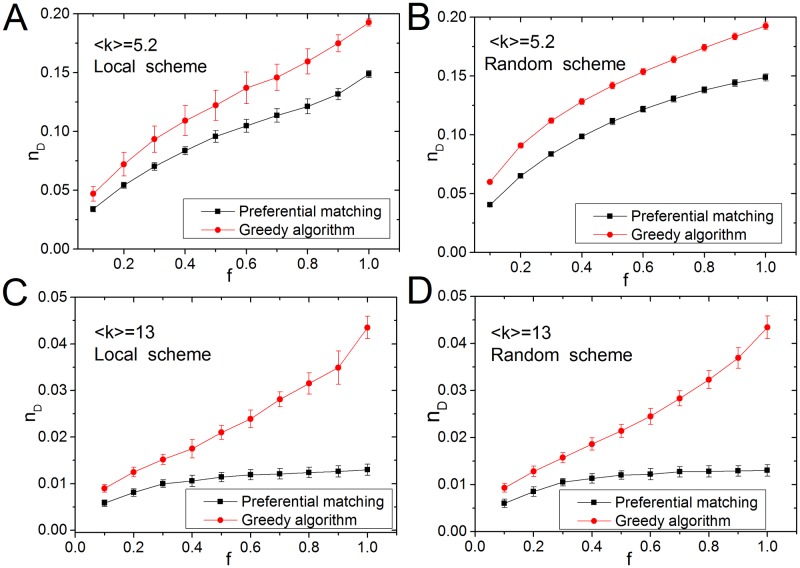
Efficiency analysis of the target control algorithm for two synthetic networks. (A-B). For the scale-free networks with *N* = 10^4^ and <*k*> = 5.2, we show the density of input nodes as a function of the fraction of target nodes. The results are computed based on 100 network instances with the same average degree. (A) Results of the local selection scheme and (B) Results of the random selection scheme. (C-D) For the scale-free networks with *N* = 10^4^ and <*k*> = 13, we show the density of input nodes as a function of the fraction of target nodes. (C) Results of the local selection scheme and (D) Results of the random selection scheme. For each network, we compute the fraction of input nodes *n*_*D*_ based on the preferential matching and the greedy algorithm. For the greedy algorithm, the *n*_*D*_ is computed based on the results of 100 random experiments.

Fig 3 shows the results of scale-free networks [16] with *N* = 10^4^. For different target node fractions *f* ∈ [0,1], the *PM* algorithm always has better performance than the *GA* in both target node selection schemes. Furthermore, the difference in the values of *n*_*D*_ obtained by *PM* and *GA*, |Δ*n*_*D*_| = |*n*_*D-*GA_- *n*_*D-*PM_|, increases with the fraction of target nodes *f*, suggesting that the *PM* algorithm is more efficient in controlling large fractions of target nodes.

**Fig 4:**
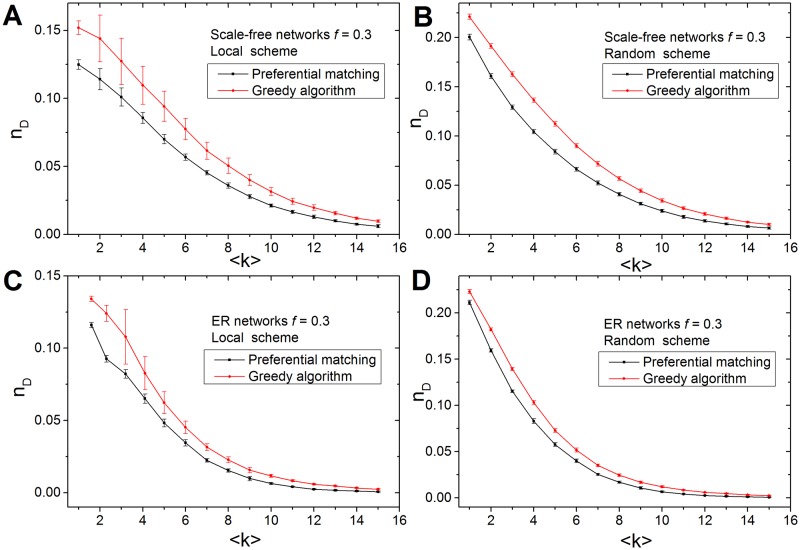
The efficiency of the algorithm for different average degree <*k*>. (A-B). For a scale-free network with *N* = 10^4^ and target node fraction *f* = 0.3, we show (A) the density of input nodes versus <*k*>, based on the local selection scheme, and (B) the density of input nodes versus <*k*>, based on the random selection scheme. (C-D). For an ER random network with *N* = 10^4^ and target node fraction *f* = 0.3, we show (C) the density of input nodes versus <*k*>, based on the local selection scheme, and (D) the density of input nodes versus <*k*>, based on the random selection scheme. For each average degree <*k*>, the fraction of input nodes *n*_*D*_ is computed based on the average results of 100 networks.

Next, we analyzed *n*_*D*_ with different average degrees <*k*>. Fig 4 shows the results for both the scale-free networks and *ER* random networks based on local and random target selection schemes. The *PM* algorithm obtains lower *n*_*D*_ than *GA* in all networks. Note that the variations of *n*_*D*_ for the local selection scheme of target nodes are much larger than those variations for the random selection scheme, suggesting that there are many input nodes set to control target nodes that are locally connected.

**Fig 5:**
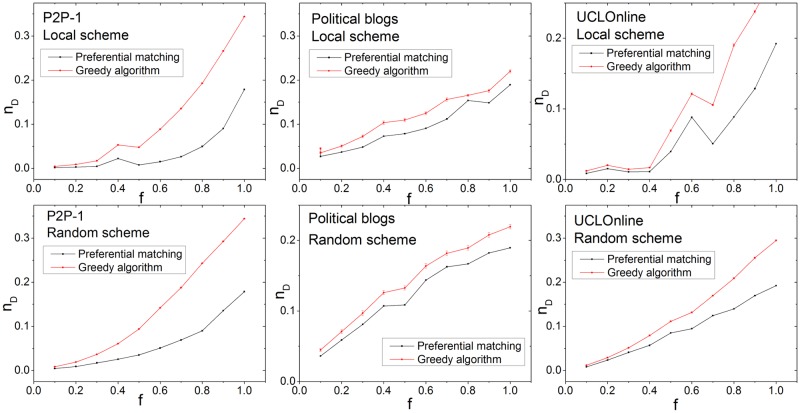
Results for real networks. We show the fraction of input nodes and the fraction of target nodes. The *PM* method always achieves better performance in both the local and random target node selection schemes.

We also evaluated the performance of the *PM* algorithm in real networks. The networks are selected based on diversity of topological structure and include food web, transcription, citation, and Internet networks. The results are shown in Table 1 and Fig 5. For all networks and fractions of target nodes in both random and local schemes, the *PM* algorithm outperforms the *GA*.

**Table 1:** Results for the real networks analyzed in the paper.

Type	Name	*N*	*L*	<*k*>	Random selection		Local selection	
					*n*_*pd*_	*n*_*rd*_	*n*_*pd*_	*n*_*rd*_
**Food Web**	Mangrove[17]	97	1492	30.76	3.09%	5.05%	4.12%	6.27%
	Silwood[18]	154	370	4.81	30.52%	31.27%	24.03%	25.37%
**Neuronal**	C. elegans[19]	306	2345	15.33	7.07%	7.97%	4.38%	5.55%
**Transcription**	E. coli[20]	423	578	2.73	39.95%	43.04%	32.39%	38.02
	TRN-Yeast-1[21]	4441	12873	5.80	45.49%	45.68%	43.75%	44.13%
	TRN-Yeast-2[22]	688	1079	3.14	39.68%	40.67%	31.40%	33.33%
**Trust**	Prison inmate[23, 24]	67	182	5.43	13.43%	17.51%	11.94%	13.84%
	WikiVote[25]	7115	103689	29.15	39.68%	40.33%	37.26%	38.37%
**Electronic circuits**	s208a[22]	122	189	3.10	9.02%	14.75%	6.56%	10.03%
	s420a[22]	252	399	3.17	8.33%	14.43%	3.57%	7.33%
	s838a[22]	512	819	3.20	6.64%	12.10%	1.95%	5.77%
**Citation**	ArXiv-HepTh[26]	27770	352807	25.41	13.41%	15.10%	7.62%	10.03%
	Kohonen[27]	4470	12731	5.70	17.90%	21.11%	9.49%	13.26%
**WWW**	Political blogs[28]	1224	16718	27.32	10.87%	13.26%	7.84%	10.99%
**Internet**	p2p-1[29]	10876	39994	7.35	3.51%	9.41%	0.77%	4.80%
	p2p-2[29]	8846	31839	7.20	4.70%	10.44%	2.32%	6.11%
	p2p-3[29]	8717	31525	7.23	4.73%	10.52%	2.44%	7.48%
**Social network**	UClonline[30]	1899	20296	21.38	8.48%	11.14%	3.95%	6.91%
	Facebook_0[31]	347	5038	29.04	1.80%	2.69%	0.30%	0.60%
	Facebook_107[31]	1912	53498	55.96	0.10%	0.19%	0.10%	0.10%
	Facebook_348[31]	572	6384	22.32	0.89%	1.79%	0.45%	0.45%

For each network, we show its type, name, number of nodes (*N*) and edges (*L*), average degree <*k*>, density of input nodes (*n*_*pd*_) based on preferential matching, and density of input nodes (*n*_*rd*_) based on random matching. The fraction of target nodes *f* = 0.5.

## Discussion

The controllability of complex networks is of great importance in many applications. Controlling a small fraction of target nodes is a common task in many real control scenarios. Here we proposed a novel algorithm based on preferential matching to reduce the number of input nodes. Our algorithm has the same main steps as the previous algorithm [13], based on multi-maximum matching of the induced bipartite graphs. However, we elaborately arranged the matching order of the nodes, which can significantly reduce the number of resulting input nodes.

However, our algorithm still cannot guarantee the optimum result. Future work should focus on finding an efficient and precise method to reduce the number of input nodes.

## Supporting information

S1 Fig Efficiency analysis of the target control algorithm for three scale-free networks with *N* = 10^4^. We show the density of input nodes as a function of the fraction of target nodes based on local and random schemes. For each network, we compute the density of input nodes *n*_*D*_ based on the preferential matching and the greedy algorithm. For the greedy algorithm, the *n*_*D*_ is computed based on the results of 100 random experiments.
(TIF)

S2 Fig The density of input nodes *n*_*D*_ versus the fraction of target nodes *f* of real networks. We show the results of ArXiv-HepTh, C.Elegans, Kohonen and Facebook_0 networks.
(TIF)

S3 Fig The density of input nodes *n*_*D*_ versus the fraction of target nodes *f* of real networks. We show the results of P2P-2, P2P-3, S208, S420 and S838 networks.
(TIF)

## References

[1] Kalman RE. Mathematical Description of Linear Dynamical Systems. Journal of the Society for Industrial & Applied Mathematics. 1963;1(2):152–192.

[2] Luenberger DG. Introduction to Dynamic Systems: Theory, Models, & Applications. Proceedings of the IEEE. 1979;69(9):1173.

[3] Slotine JJE, Li W. Applied nonlinear control. Beijing: China Machine Press; 2004.

[4] Zhang X, Lv T, Pu Y. Input graph: the hidden geometry in controlling complex networks. Scientific Reports. 2016;6:38209 10.1038/srep38209 27901102 PMC5128914

[5] Ruths J, Ruths D. Control profiles of complex networks. Science. 2014;343(6177):1373–1376. 10.1126/science.1242063 24653036

[6] Liu YY, Slotine JJ, Barabasi AL. Controllability of complex networks. Nature. 2011;473(7346):167–173. 10.1038/nature10011 21562557

[7] Murota K. Matrices and Matroids for Systems Analysis. Berlin: Springer Science & Business Media; 2000.

[8] Jia T, Barabási AL. Control capacity and a random sampling method in exploring controllability of complex networks. Scientific Reports. 2013;3:2354 10.1038/srep02354 23912679 PMC3733055

[9] Jia T, Posfai M. Connecting core percolation and controllability of complex networks. Scientific Reports. 2014;4:5379 10.1038/srep05379 24946797 PMC4064349

[10] Pósfai M, Liu YY, Slotine JJ, Barabási AL. Effect of correlations on network controllability. Scientific Reports. 2012;3:1067. 10.1038/srep01067 PMC3545232 23323210

[11] Pu CL, Pei WJ, Michaelson A. Robustness analysis of network controllability. Physica A Statistical Mechanics & Its Applications. 2012;391(18):4420–4425.

[12] Jia T, Liu YY, Csóka E, Pósfai M, Slotine JJ, Barabási AL. Emergence of bimodality in controlling complex networks. Nature Communications. 2013;4:2002 10.1038/ncomms3002 23774965

[13] Gao J, Liu YY, D'souza RM, Barabási AL. Target control of complex networks. Nature Communications. 2014;5:5415 10.1038/ncomms6415 25388503 PMC4243219

[14] Piao X, Lv T, Zhang X, Ma H. Strategy for community control of complex networks. Physica A Statistical Mechanics & Its Applications. 2015;421:98–108.

[15] Zhang X, Lv T, Yang XY, Zhang B. Structural controllability of complex networks based on preferential matching. PLoS One. 2014;9(11):e112039 10.1371/journal.pone.0112039 25375628 PMC4222963

[16] Barabási AL, Albert R. Emergence of scaling in random networks. Science. 1999;286(5439):509–512. 10521342 10.1126/science.286.5439.509

[17] Ulanowicz RE, DeAngelis DL. Network analysis of trophic dynamics in south florida ecosystems. US Geological Survey Program on the South Florida Ecosystem. 2005;114.

[18] Montoya JM, Solé RV. Small world patterns in food webs. Journal of Theoretical Biology. 2002;214(3):405–412. 10.1006/jtbi.2001.2460 11846598

[19] Watts DJ, Strogatz SH. Collective dynamics of ‘small-world’networks. Nature. 1998;393(6684):440–2. 10.1038/30918 9623998

[20] Shen-Orr S, Milo R, Mangan S, Alon U. Network motifs in the transcriptional regulation network of Escherichia coli. Nature Genetics. 2002;31(1):64–68. 10.1038/ng881 11967538

[21] Bu D, Zhao Y, Cai L, Xue H, Zhu X, Lu H, et al Topological structure analysis of the protein–protein interaction network in budding yeast. Nucleic acids research. 2003;31(9):2443–2450. 12711690 10.1093/nar/gkg340 PMC154226

[22] Milo R, Shen-Orr S, Itzkovitz S, Kashtan N, Chklovskii DB, Alon U. Network motifs: Simple building blocks of complex networks. Science. 2002;42(6821):285–298. 10.1126/science.298.5594.824 12399590

[23] Milo R, Itzkovitz S, Kashtan N, Levitt R, Shen-Orr S, Ayzenshtat I, et al Superfamilies of evolved and designed networks. Science. 2004;303(5663):1538–1542. 10.1126/science.1089167 15001784

[24] Van Duijn MAJ, Zeggelink EPH, Huisman M, Stokman F, Wasseur FW. Evolution of sociology freshmen into a friendship network. Journal of Mathematical Sociology. 2003;27(2–3):153–191.

[25] Leskovec J, Lang KJ, Dasgupta A, Mahoney MW. Community structure in large networks: Natural cluster sizes and the absence of large well-defined clusters. Internet Mathematics. 2009;6(1):29–123.

[26] Leskovec J, Kleinberg J, Faloutsos C. Graphs over time: densification laws, shrinking diameters and possible explanations. Proceedings of the eleventh ACM SIGKDD international conference on Knowledge discovery in data mining; 2005: 177–187.

[27] Handcock MS, Hunter D, Butts CT, Goodreau SM, Morris M. Statnet: An R package for the Statistical Modeling of Social Networks. 2003. http://www.csde.washington.edu/statnet.

[28] Adamic LA, Glance N. The political blogosphere and the 2004 US election: divided they blog. Proceedings of the 3rd international workshop on Link discovery; 2005: 36–43.

[29] Leskovec J, Kleinberg J, Faloutsos C. Graph evolution: Densification and shrinking diameters. ACM Transactions on Knowledge Discovery from Data (TKDD). 2007;1(1):2–41.

[30] Opsahl T, Panzarasa P. Clustering in weighted networks. Social Networks. 2009;31(2):155–163.

[31] Mcauley JJ, Leskovec J. Learning to discover social circles in ego networks. Advances in Neural Information Processing Systems; 2012:539–547.

