# Supplementary figures and images for: Efficient target control of complex networks based on preferential matching
Source: PLoS One. 2017 Apr 6;12(4):e0175375. doi: 10.1371/journal.pone.0175375 (PMC5383299; doi:10.1371/journal.pone.0175375)

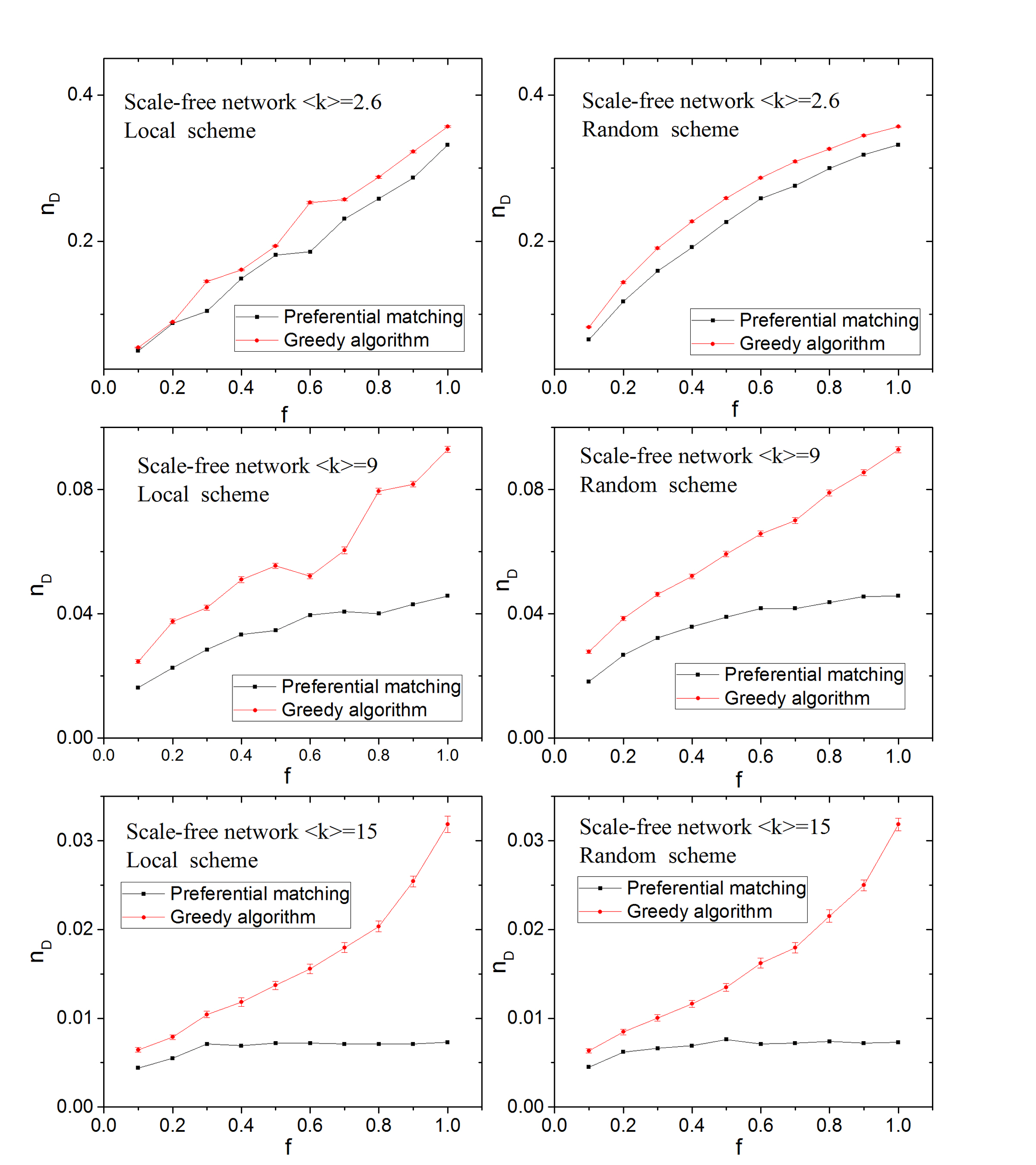

Supplement: S1 Fig — We show the density of input nodes as a function of the fraction of target nodes based on local and random schemes. For each network, we compute the density of input nodes nD based on the preferential matching and the greedy algorithm. For the greedy algorithm, the nD is computed based on the results of 100 random experiments. (TIF) [file pone.0175375.s001.tif]

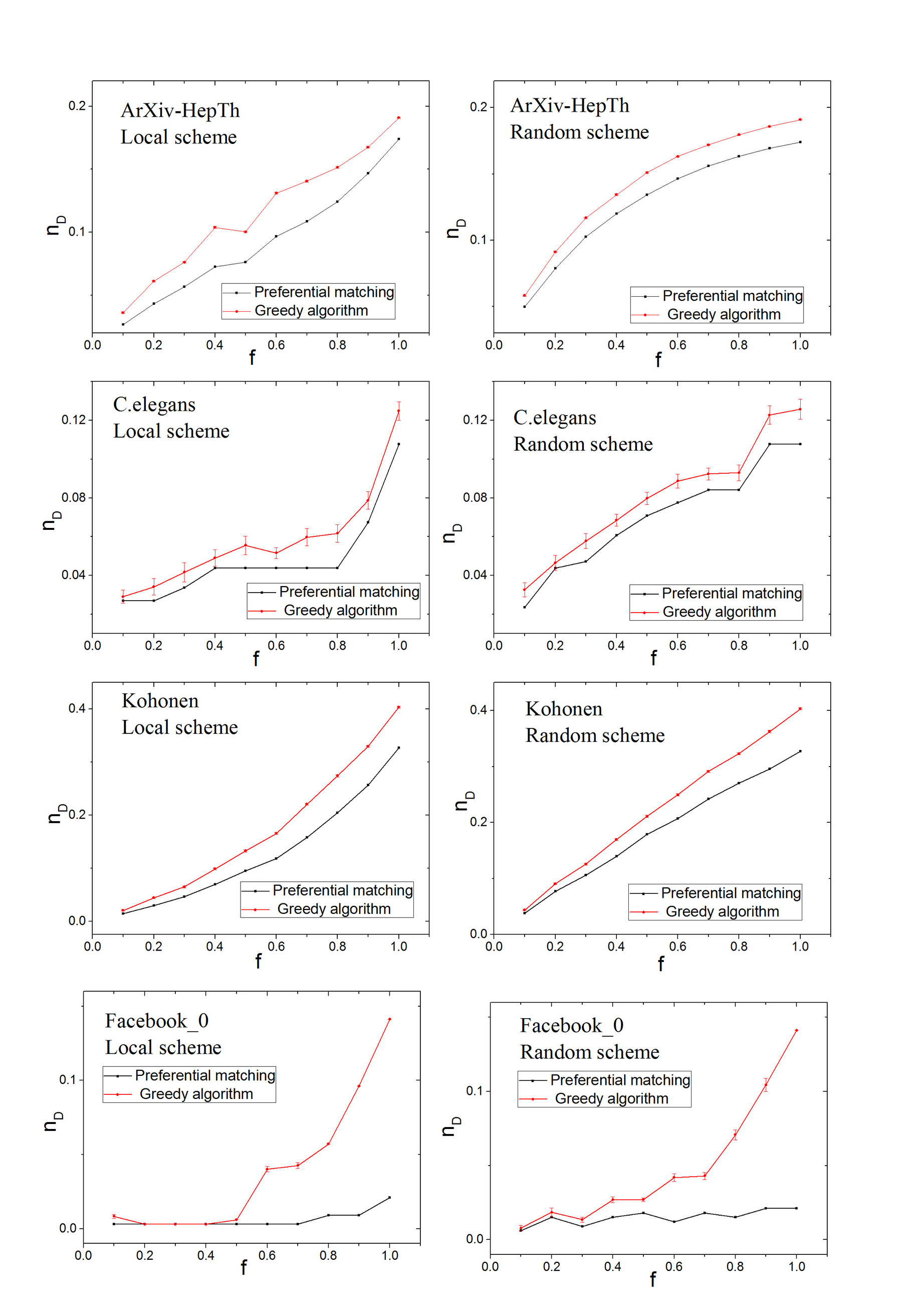

Supplement: S2 Fig — We show the results of ArXiv-HepTh, C.Elegans, Kohonen and Facebook_0 networks. (TIF) [file pone.0175375.s002.tif]

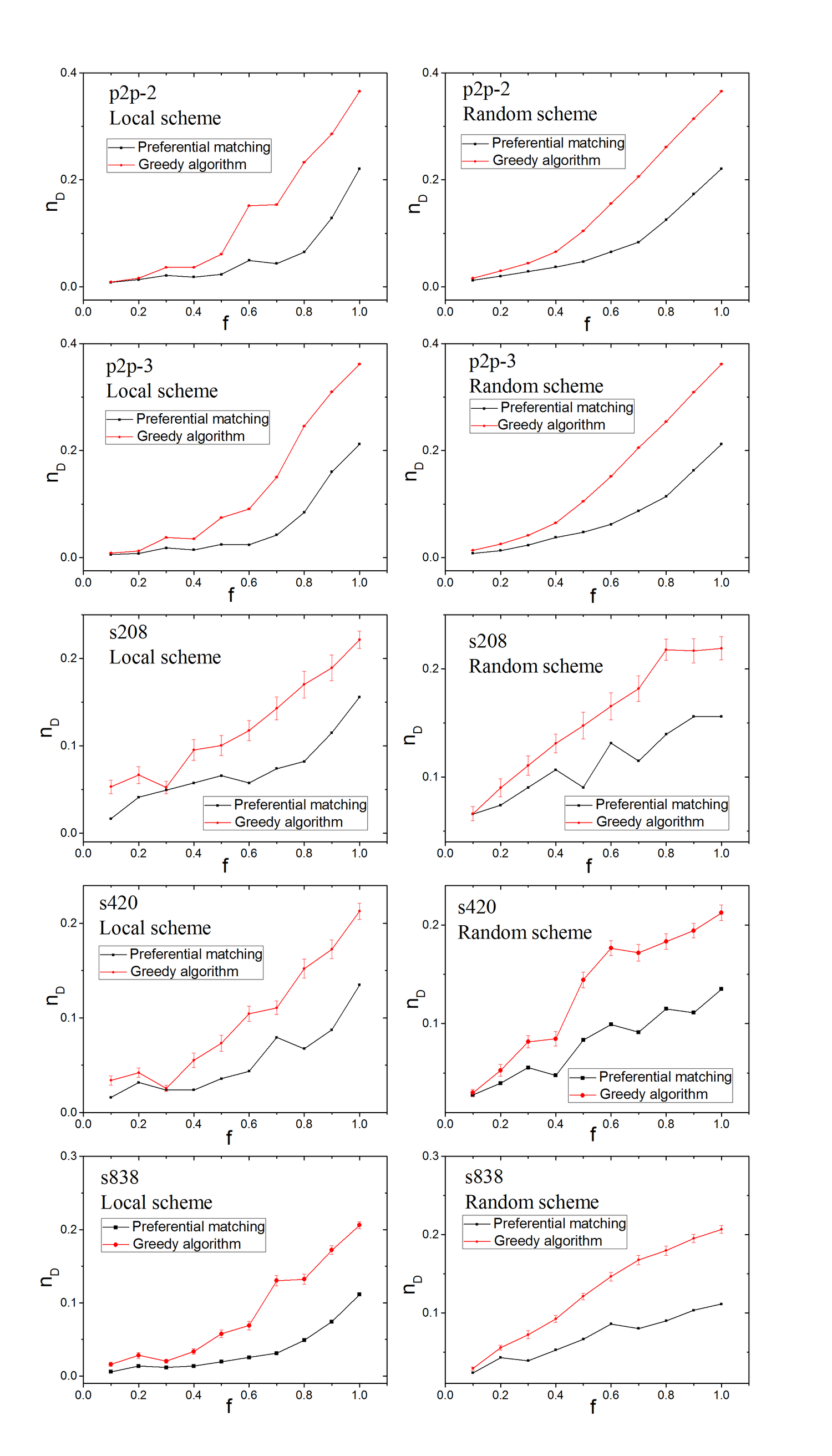

Supplement: S3 Fig — We show the results of P2P-2, P2P-3, S208, S420 and S838 networks. (TIF) [file pone.0175375.s003.tif]
